# Novel Nanozeolitic Imidazolate Framework (ZIF-8)–Luciferase Biocomposite for Nanosensing Applications

**DOI:** 10.1021/acs.analchem.2c05001

**Published:** 2022-12-06

**Authors:** Héctor Martínez-Pérez-Cejuela, Denise Gregucci, Maria Maddalena Calabretta, Ernesto Francisco Simó-Alfonso, José Manuel Herrero-Martínez, Elisa Michelini

**Affiliations:** † Department of Chemistry “Giacomo Ciamician”, University of Bologna, Via Selmi 2, 40126 Bologna, Italy; ‡ Department of Analytical Chemistry, University of Valencia, C/Dr. Moliner, 50, 46100 Burjassot, Valencia, Spain; § Center for Applied Biomedical Research (CRBA), Azienda Ospedaliero-Universitaria Policlinico S. Orsola-Malpighi, 40138 Bologna, Italy; ∥ Health Sciences and Technologies Interdepartmental Center for Industrial Research (HSTICIR), University of Bologna, 40126 Bologna, Italy

## Abstract

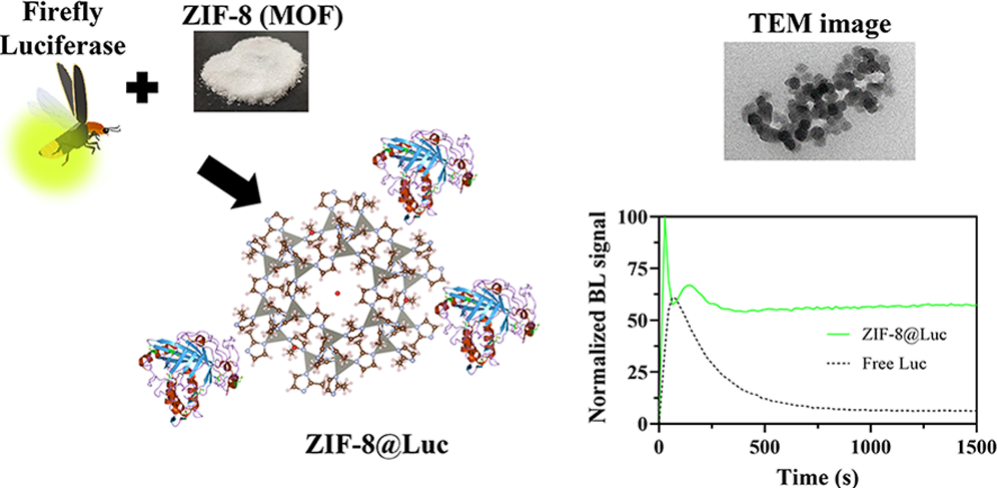

The identification of new strategies to improve the stability of proteins is of utmost importance for a number of applications, from biosensing to biocatalysis. Metal–organic frameworks (MOFs) have been shown as a versatile host platform for the immobilization of proteins, with the potential to protect proteins in harsh conditions. In this work, a new thermostable luciferase mutant has been selected as a bioluminescent protein model to investigate the suitability of MOFs to improve its stability and prompt its applications in real-world applications, for example, ATP detection in portable systems. The luciferase has been immobilized onto zeolitic imidazolate framework-8 (ZIF-8) to obtain a bioluminescent biocomposite with enhanced performance. The biocomposite ZIF-8@luc has been characterized in harsh conditions (e.g., high temperature, non-native pH, etc.). Bioluminescence properties confirmed that MOF enhanced the luciferase stability at acidic pH, in the presence of organic solvents, and at −20 °C. To assess the feasibility of this approach, the recyclability, storage stability, precision, and Michaelis–Menten constants (*K*_m_) for ATP and d-luciferin have been also evaluated. As a proof of principle, the suitability for ATP detection was investigated and the biocomposite outperformed the free enzyme in the same experimental conditions, achieving a limit of detection for ATP down to 0.2 fmol.

Bioluminescence (BL) can be defined as the emission of light due to a chemical reaction occurring in a living organism. This phenomenon has been exploited as a powerful tool in biological and chemical sciences.^1,2^*Photinus pyralis* luciferase, thanks to its high quantum yield (0.44) is one of the most studied BL enzymes.^3,4^ Although firefly species with different origins and emission properties have been described, most of them have similar bioluminescent chemical systems sharing the same substrates (d-luciferin, ATP, and Mg(II)).^3,5^ The extensive characterization of these enzymes enabled their application in several fields such as ATP determination,^6^ microbial detection,^7^ reporter gene assays,^8^ and biosensing.^9^

Despite the high number of reports described in the literature, few of them found practical application. It is known that practical applications of luciferases are hindered by their delicate nature against harsh conditions (e.g., temperature, pH, chemical agents, etc.).^10,11^ This hampers the development of luciferase-based biosensors for on-field applications. To this end, many efforts have been done in the last two decades to obtain highly stable luciferases. For example, random and site-directed mutagenesis studies of the luciferase gene have been widely explored for enhancing its stability and catalytic activity.^12,13^ Branchini et al. obtained several *P. pyralis* luciferase mutants and used them for ATP determination in cellular environments with high sensitivity.^14^ Further genetic changes to luciferases have been performed to obtain thermostable and pH-resistant luciferase mutants and variants emitting at different wavelengths.^2,15^ Although this possibility is useful and very interesting, mutagenesis studies require great expertise in the field as well as well-equipped laboratories. As an alternative or synergic strategy, the immobilization of luciferase onto nanomaterials and functional materials has been also explored to enhance the performance and stability of the luciferase.^11,16^ Some studies have already reported the use of different materials for attaching luciferases, such as agarose polymers,^17^ graphite platforms,^18^ nanofiber membranes,^19^ and silica.^20,21^ However, these methods present some important drawbacks including luciferase inactivation or significant decrease of its catalytic activity,^18,20^ long-time preparation procedures,^19,21^ and lack of reproducibility. Therefore, there is a necessity of developing new methods to immobilize and stabilize luciferase onto functional materials to enable its use for robust and sensitive biosensing. To date, there are two main approaches for enzyme immobilization: (i) via adsorption, which is simple and straightforward, allowing the exposition to the media, and (ii) the encapsulation by one-pot synthesis, which normally provides better functionality under general conditions (for instance, protected environment). Both present benefits and drawbacks and the selection should be done taking into account the final application.^22,23^

Metal–organic frameworks (MOFs) are a class of microporous materials composed by the coordination of metal ions and organic ligands.^24^ MOFs, thanks to their appealing features, such as rich chemical surface, biocompatibility, and good stability,^25^ have shown promising results in the bioanalytical field, especially for biosensing, bioimaging, biomedical applications, and bioremediation applications.^26−28^ Despite the benefits of these materials as host platforms for enzymes,^10,29^ the combination with luciferase has been seldom explored.^30,31^ A very preliminary study was reported, in which luciferase was immobilized onto MIL-53(Al) and NH_2_-MIL-88(Fe) via covalent and noncovalent bindings. Those approaches provided a significant improvement in luciferase stability; however, no full characterization was obtained in terms of thermal stability, storage influence, and potential reusability and applicability (e.g., in terms of limit of detection (LOD) or precision).

Hence, in this work, we report for the first time a novel BL biocomposite in which a firefly luciferase mutant has been attached to a metal–organic framework, ZIF-8, belonging to the zeolitic imidazolate framework family. Different MOFs were investigated and ZIF-8 was selected due to its advantageous properties.^32,33^ The synthesis was optimized and neither additional reagents nor stabilizers were used in the attachment process to make the optimized synthesis process simple, straightforward, and cost-effective. The potential of the synthesized ZIF-8@Luc biocomposite has been demonstrated with an exhaustive characterization of the BL behavior with and without MOF. The combination of ZIF-8 and the new luciferase mutant with improved stability provided an enhancement of the enzymatic catalysis as well as stabilization in harsh conditions. The Michaelis–Menten constants (*K*_m_) for both ATP and d-luciferin (d-LH_2_) were studied to assess the enzyme functionality after the immobilization process. As a proof of principle, the suitability for ATP detection was investigated and the biocomposite outperformed the free enzyme in the same experimental conditions, achieving a limit of detection for ATP down to 0.2 fmol.

## Materials and Methods

Reagents and materials, instrumentation, and solution preparation can be found in the Supporting Information. Furthermore, the procedures to synthesize the bare MOFs are also summarized in this file together with characterization studies (Figures S1 and S2 and Table S1).

### General Procedures

Concentrations of purified proteins were determined with the Bio-Rad Protein Assay System using Bovine Serum Albumin (BSA) as the standard reagent. Luciferase mutant,^34^ containing the mutations F14R, L35Q, V182K, I232K, F465R Y33N, T214A, A215L, F295L, E354K, V241I, G246A, F250S, N119G, and N50D, was expressed and purified according to a previous report.^35^ The BL assay was performed in a white 384-well plate (Greiner Bio One North America, Monroe, NC). In general, each analysis was performed at least in duplicate and repeated at least two times unless otherwise stated. In a typical analysis, a 6 μL volume of 0.1 mg/mL luciferase solution/ZIF-8@Luc dispersion was dispensed in a well of a 96-well microplate with 6 μL of 1 mM d-LH_2_, 5 μL of 10 mM MgCl_2_, and 5 μL of 2 mM ATP. d-LH_2_ was added with the automatic injector. The BL emissions were recorded after a 1 s delay following injection of d-LH_2_ (25 min, integration time 500 ms) with a luminometer (Thermo Scientific Varioskan LUX Multimode microplate reader).

### Synthesis of ZIF-8@Luc and Method Optimization

The synthesis of the biocomposite was accomplished by taking the method described by Nowroozi-Nejad et al.^31^ as the starting point with several modifications and parameter optimization. The following conditions were studied in detail: type of stirring (magnetic, orbital, vortex-assisted), MOF nature (MIL-*n*, UiO-*n*, ZIF-*n*), amount of MOF (0.25–0.75 mg), and reaction time (15–60 min). The optimized synthesis was as follows: 0.25 mg of ZIF-8 was weighed in 1.5 mL Eppendorf tubes and dispersed in 90 μL of Tris–HCl (50 mM, pH 7.8) for 15 min in the ultrasonic bath. The dispersion was refrigerated in an ice bath (60 s) before adding 10 μL of luciferase (1 mg/mL). Then, the dispersion was placed in an ice bath (4 ± 1 °C) and incubated for 30 min with orbital shaking (140 rpm). After centrifuging at 18 000*g* for 5 min, the supernatant BL was measured. The pellet was washed twice with 50 μL of Tris–HCl (50 mM, pH 7.8) and resuspended in 100 μL of Tris–HCl (50 mM, pH 7.8) and homogenized with a vortex (60 s) and ultrasonic bath (120 s) before BL signal acquisition, as described in the General Procedures section. Once the synthesis was optimized, emission kinetics and spectra were recorded by concentrating the ZIF-8@Luc biocomposite using a lower redispersion volume (20 μL) (∼0.5 mg/mL luciferase conc.).

### Performance of ZIF-8@Luc in Harsh Conditions

The resulting ZIF-8@Luc was extensively studied in different harsh conditions. In particular, the pH stability of ZIF-8@Luc was analyzed by adjusting the pH of 50 mM Tris–HCl to 5.0, 8.0, and 10.0, respectively. A 6 μL volume of a buffer solution was added to 6 μL of the enzyme solution with and without MOF to adjust the final pH to 5.0, 8.0, and 10.0. The mixture was incubated for 5 min before BL signal was acquired. Temperature resistance was assessed at different temperatures (−20 and 50 °C) for 2 h. In both cases, the temperature was set at 4 °C for 2 min prior to the BL signal acquisition. The dryness/reconstitution process was performed as follows: for the biocomposite, the dispersion was centrifuged and the supernatant was discarded. Then, the pellet was dried at 25 °C overnight. In the case of free luciferase, a small volume (for instance, 15 μL) was dried at 25 °C overnight without the centrifugation step. The following day, both dried pellets were redispersed in the same Tris–HCl volume. Lastly, the influence of organic solvents on the BL response was studied using 5 min incubation with four solvents (acetone, acetonitrile, ethanol, and isopropanol) with the ratio 1:1 (v/v) between ZIF-8@Luc/free luciferase and organic solvent (same procedure described in pH stability studies).

### Characterization of ZIF-8@Luc and Kinetic Studies

Regarding the recyclability study, ZIF-8@Luc was synthesized as described previously. Then, 100 μL of 1 mM d-LH_2_, 83 μL of 10 mM of Mg(II), and 83 μL of 2 mM ATP were added to the Eppendorf tube. A quick homogenization was performed with the vortex (with 15 s gentle stirring) and two BL measurements were performed adding 25 μL of the dispersion. After the signal collection, the solution was mixed again and centrifuged for 5 min at 18 000*g*. Next, the supernatant was discarded, and the pellet was washed twice with 50 μL of Tris–HCl. The process was performed in duplicate and repeated four times. For storage stability, two syntheses were performed, and the resulting dispersions were sealed and stored at 4 and 25 °C in the darkness. The *K*_m_ was determined using saturating levels of d-LH_2_ (0.001–5 mM) and ATP (from 10^–8^ to 10^2^ mM). The assays were performed in triplicate. The precision of the BL signal in the ATP studies was assessed with different concentrations at different times.

### Proof of Principle: ATP Quantification

A stock solution of ATP (20 mM) in 50 mM Tris–HCl (pH 7.8) was used to prepare ATP solutions in the range from 10^–8^ to 10^2^ mM. The BL signal was recorded for 30 min. The ATP dose–response curve was carried out by calculating the intensity mean of four different measurements at 20 min (with the biocomposite and free luciferase). LODs were calculated as the blank plus 3 times the standard deviation of four replicates.

## Results and Discussion

### Rationale and Design of the Luciferase–MOF Biocomposite

Prompted by the need of improving the luciferase stability for real-world biosensing applications, we explored the use of MOFs with a newly developed thermostable luciferase. The *P. pyralis* luciferase gene was mutated to improve the pH and thermostability by applying previously reported mutations, which showed to increase the BL emission of the wild-type luciferase at pH 6.0 by 2 times and increase the stability of the protein at a higher temperature. We included mutations F14R, L35Q, V182K, I232K, and F465R, which do not change the *K*_m_ for ATP and d-LH_2_,^36^ and added mutation Y33N, not yet reported, and mutations T214A, A215L, F295L, E354K, V241I, G246A, N119G, N50D, and F250S, to improve *in vitro* stability and *in vivo* sensitivity.^37,38^

Prior to luciferase attachment to the MOF, some considerations have to be taken into account, such as (i) the size of the enzyme (60 kDa) and MOF pore cavities (3.4 Å);^39^ (ii) the selection of nontoxic MOFs; and (iii) the method for enzyme incorporation has to be optimized to maintain the structure of both MOF and protein. Preliminary studies were thus conducted to select the best MOF carrier. Three MOFs were selected as starting materials to perform the attachment. ZIF-8, due to its well-demonstrated capability as a carrier for different biomolecules;^32^ UiO-66 and MIL-101(Al), thanks to their high biocompatibility.^31,40^

We preliminary investigated the BL signal of a mixture of luciferase solutions with and without MOF dispersions to assess the potential negative effects of the MOFs on BL emission (Figure S3). The presence of MOFs, even at high concentrations (80 mg/L) did not affect the signal compared to the luciferase in the same experimental conditions. These findings could be explained by the lack of interaction between MOFs and luciferase. We performed luciferase attachment onto three selected MOFs (ZIF-8, MIL-101(Al), and UiO-66) (Figure S4). The surface attachment was selected due mainly to two factors: (i) it is the most straightforward method and (ii) it allows the operation in mild conditions, which is essential for the preservation of the component integrities.^10^

UiO-66 and MIL-101(Al) caused almost complete inhibition of the enzyme catalytic activity with a ∼98% decrease of the BL signal (Figure S4), while ZIF-8 caused about an 85% decrease of the BL signal. A possible explanation could be the large capacity and chemical stability of ZIF-8 in physiological environments,^32^ but also the higher toxicity (e.g., due to the generation of reactive oxygen species) of Zr (IV) and Al (III) compared to Zn (II).^41^ Therefore, ZIF-8 was selected for further studies.

**Figure 1:**
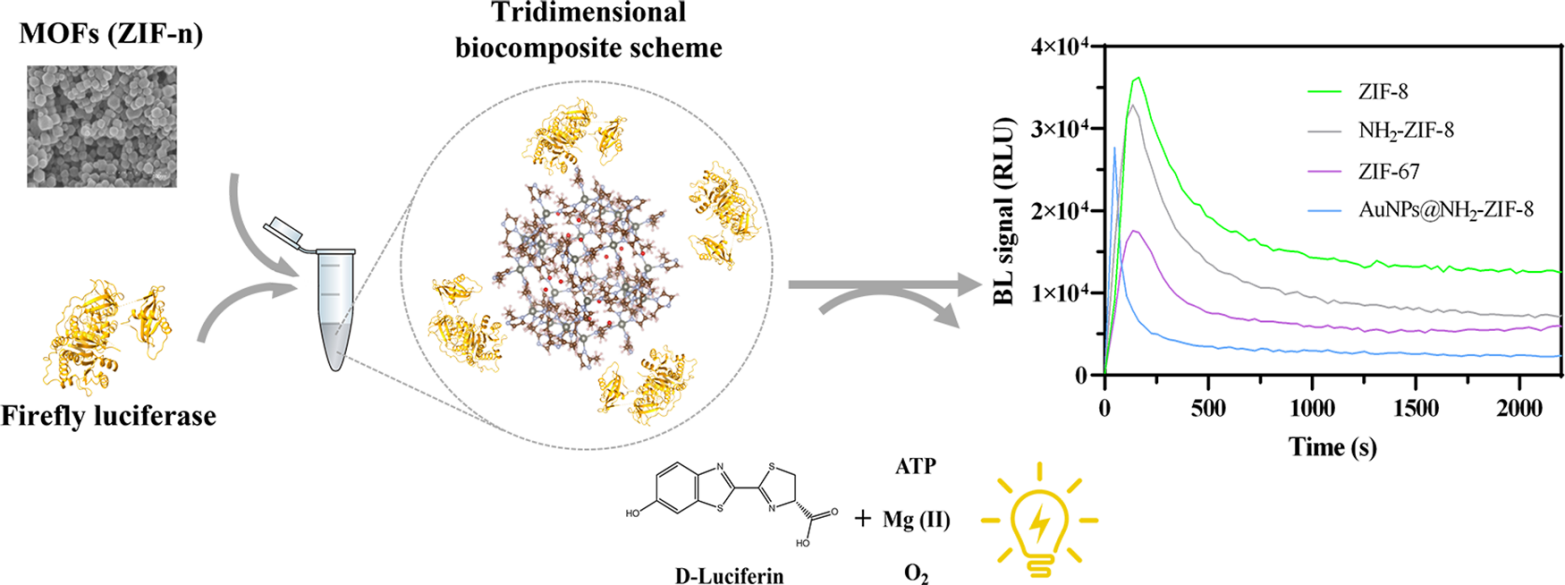
Schematic representation of the MOF-based bioluminescent biocomposite and BL kinetic measurements of the different ZIF-n@Luc biocomposites.

Different MOFs belonging to the ZIF family^39,42,43^ were studied to compare their performance. Several candidates were selected including NH_2_-ZIF-8, AuNP@NH_2_@ZIF-8, and ZIF-67, and the attachment was performed under the same experimental conditions. ZIF-8 was the best material for luciferase attachment in terms of BL signal intensity (Figure 1), with a BL signal of 1.4 × 10^4^ relative luminescent unit (RLU) at 600 s. When compared to their amino homologues, NH_2_-ZIF-8 and the gold-derived version AuNP@NH_2_@ZIF-8 caused 36 and 78% BL signal decreases with BL emissions of 9.0 × 10^3^ and 3.2 × 10^3^ RLU, respectively. The low surface availability of modified ZIF-8 could be a problem in the subsequent luciferase attachment, but also the introduction of Au(0) or Co(II) could produce an inhibition on the luciferase activity. ZIF-8 material was thus selected.

### Interactions between ZIF-8 and Luciferase

Due to the large and complex structure of the luciferase, multiple and diverse types of interactions occur with ZIF-8. Although further studies will be required for understanding the type of bindings, preliminary speculations can hypothesize the formation of hydrogen bonding between luciferase surface-exposed amino acids and the nitrogen present in 2-methyl imidazole (HMIM). These free amino groups can coordinate with Zn(II). A lower contribution could be expected in the formation of covalent peptide bonds from the nitrogen heteroatom in the HMIM ligand and the free carboxylate groups from the enzyme surface. It is also worth mentioning that imidazole compound is used after the purification process to stabilize the luciferase and preserve its functionality; therefore, HMIM can further stabilize the enzyme attached to the surface (this hypothesis can support also data shown in Figure S4). The contribution of hydrophobic interactions (e.g., van der Waals forces) and Π-stacking could be also relevant; ZIF-8 imidazole ring can interact with luciferase hydrophobic regions (i.e., phenyl groups of amino acid residues). The displacement of water molecules from the surfaces and, subsequently, the entropy gain^11^ makes nonspecific interactions one of the most important contributions to the attachment. The attachment of the ZIF-8@Luc was assessed by Fourier transform infrared (FT-IR) (Figure S5), confirming the perfect integrity of both components since all of the bands from ZIF-8 were present in the biocomposite as well as a small peak present at 1075 cm^–1^ from luciferase. Computational studies will be required to corroborate the major contribution to this biocomposite.

### Characterization of ZIF-8

**Figure 2:**
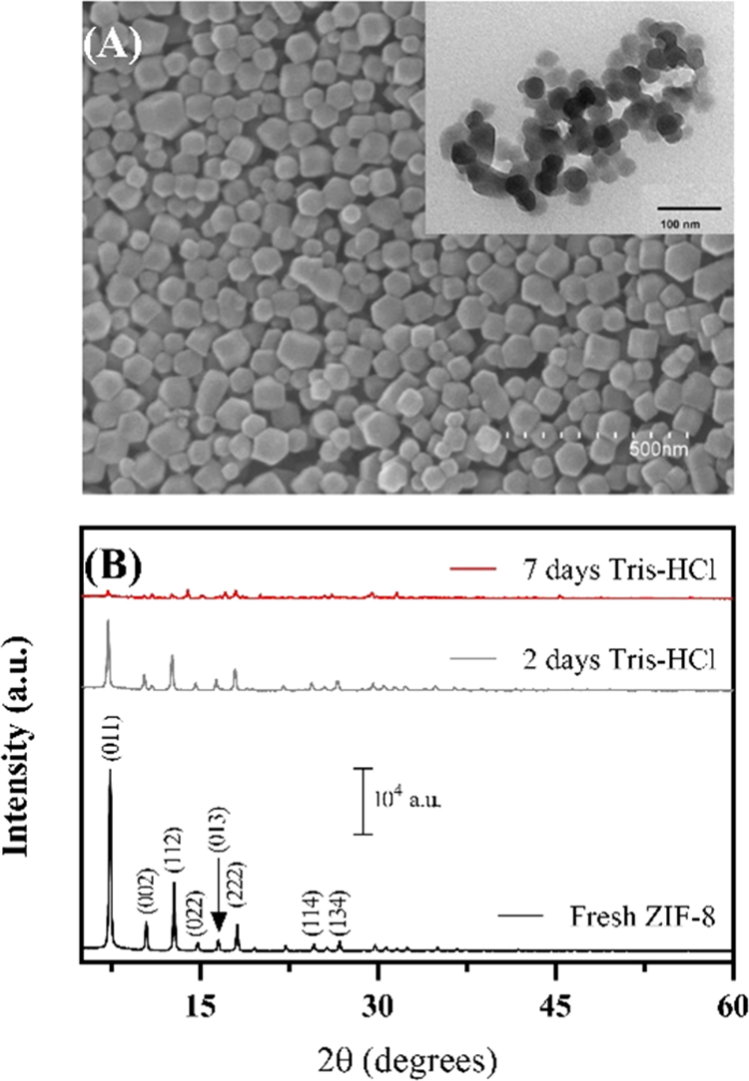
ZIF-8 characterization studies. (A) Main image represents the SEM micrograph and the inset represents the TEM micrograph. (B) p-XRD analysis of the fresh ZIF-8 (black line) and after its immersion in Tris–HCl for 2 days (gray) and 7 days (red), respectively.

The results from scanning and transmission electron microscopies (SEM and TEM, respectively) are shown in Figure 2A. The predominant morphology is the typical polyhedral shape characteristic of the pristine ZIF-8^39^ with an average diameter size of 64 ± 8 nm (*n* = 50). The surface area was also evaluated by Brunauer–Emmett–Teller (BET) surface area analysis (Table S1), obtaining more than 1000 m^2^/g for ZIF-8.

Powder X-ray diffraction (p-XRD) spectra from some ZIF-8 and the other MOFs (Figure S1) samples were also obtained (Figure 2B). ZIF-8 spectrum has the typical diffraction peaks corresponding to its structure,^39^ suggesting that the typical sodalite structure of ZIF-8 is well-formed. Furthermore, the ZIF-8 spectra were recorded in 50 mM Tris–HCl pH 7.8 for 2 and 7 days at room temperature. Meanwhile, significant structure breakdown was observed after 7 days, and only partial degradation was observed after 2 days, in accordance with other studies.^44^ These results were further confirmed with the FT-IR spectra (Figure S2). The same trend was observed in this case, confirming the importance of solvent selection for further studies.

### Synthesis Optimization

After performing the characterization and the preliminary studies, different parameters of the synthesis process were optimized (Figures S6 and S7A,B). The best experimental conditions were as follows: (i) type of stirring: orbital; (ii) ZIF-8 amount: 0.25 mg; and (iii) synthesis time: 30 min. Further discussion can be found in the Supporting Information, which explains the low BL signals caused by magnetic stirring. Luciferase leaking was also studied by performing several washing steps with Tris buffer (3 × 100 μL). Figure S8 clearly evidences that there was no loss of enzyme activity after the binding. Furthermore, the loading capacity was established as 400 μg/mg since the binding efficiency is nearly 100%.

**Figure 3:**
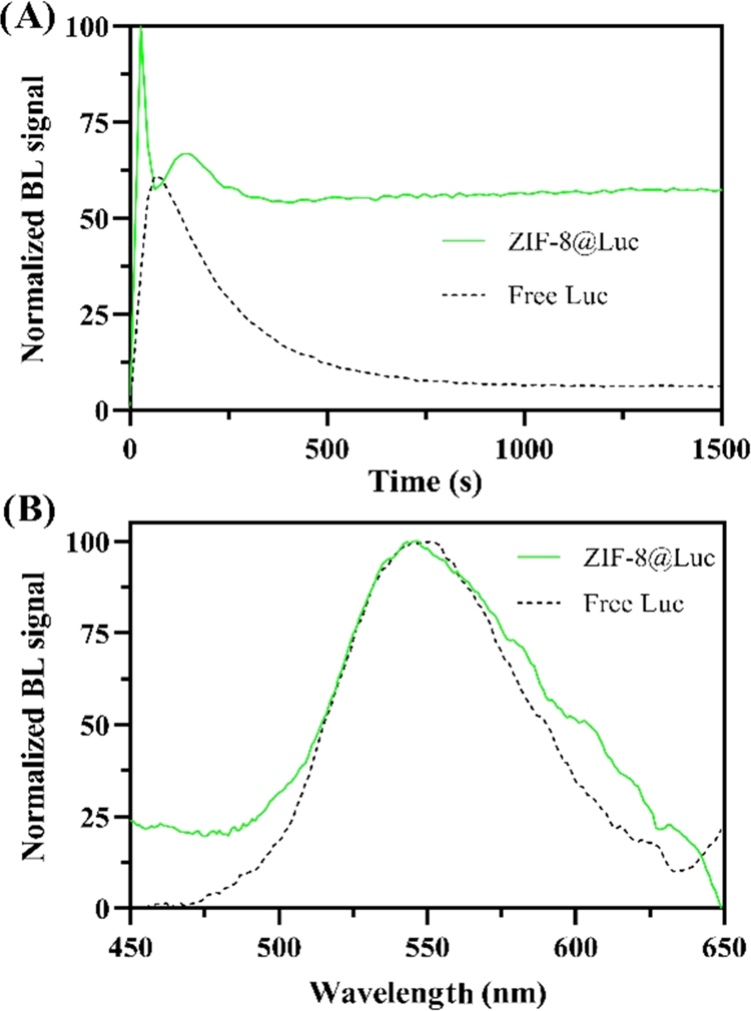
Characterization of ZIF-8@Luc biocomposite and free luciferase. (A) BL emission kinetics for 25 min after the automatic injection of d-LH_2_. (B) BL emission spectra obtained with 0.5 mg/mL luciferase in both cases.

After synthesis optimization, the characterization of ZIF-8@Luc in terms of kinetic measurement and emission spectrum was obtained (Figure 3). The attachment of luciferase onto ZIF-8 provided better signal stabilization over time (>10 min) and a slight signal enhancement in the first 2 min in the tested conditions (40% enhancement) (Figure 3A). These findings are very interesting because (i) ZIF-8@Luc has shown higher sensitivity, as already reported with fluorescent systems,^45^ and (ii) the glow-type kinetics enables to perform the measure with luminometers not equipped with automatic injectors, probably due to the self-assembled conformation of ZIF-8@Luc.^46^ In the same conditions, BL emission maxima of 544 and 551 nm were obtained with the luciferase and ZIF-8@Luc with a half bandwidth of 75 and 86 nm, respectively (Figure 3B).

### Performance of ZIF-8@Luc in Harsh Conditions

**Figure 4:**
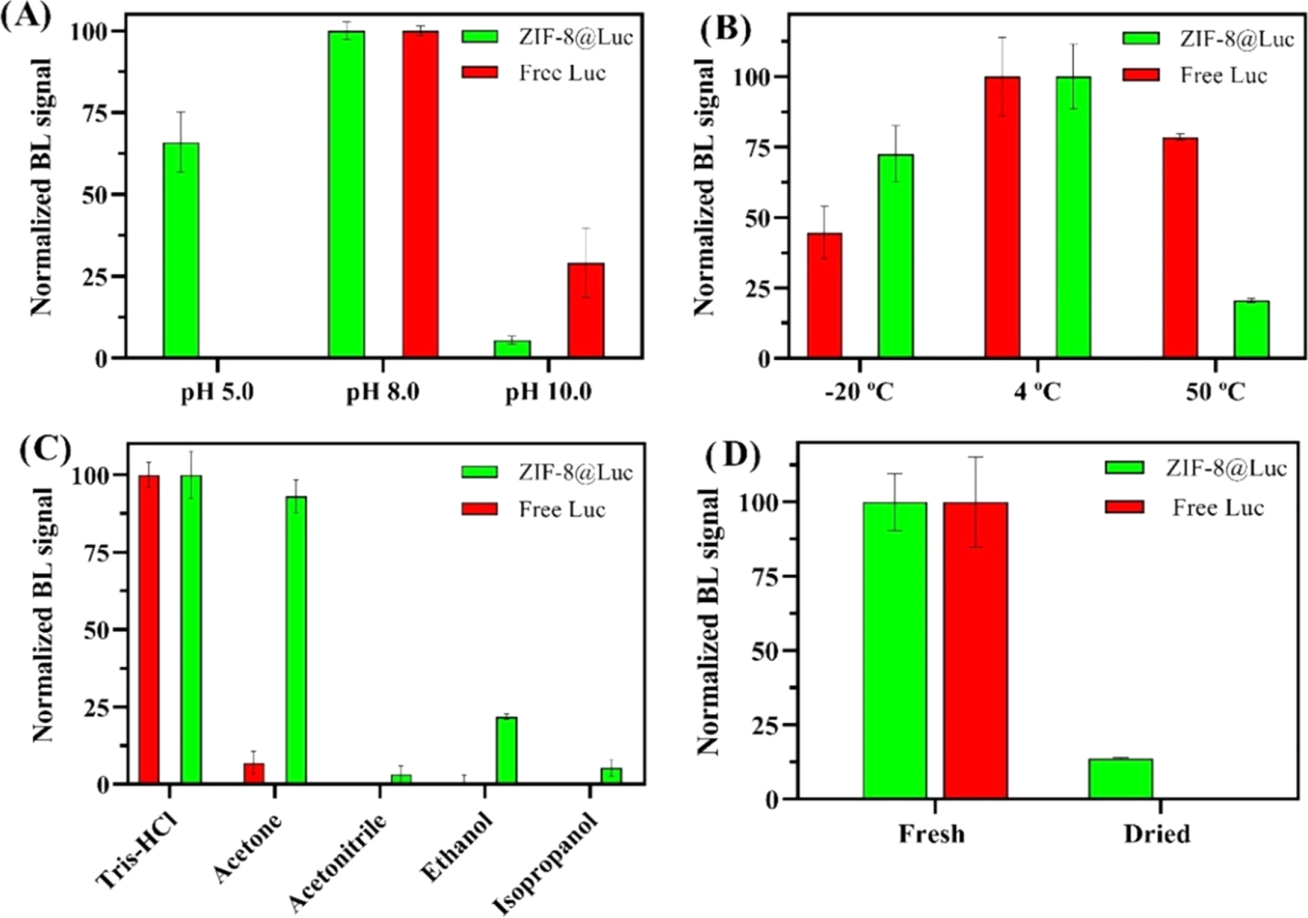
Stability studies in terms of BL response using the optimized ZIF-8@Luc and free luciferase (A) pH study for 5 min buffering from 5.0 to 10.0. (B) Temperature study from −20 to 50 °C for 2 h. (C) Resistance to the presence of organic solvents (50:50, v/v) for 5 min using acetone, acetonitrile, ethanol, and isopropanol. (D) Drying the biocomposite overnight and redispersing the pellet at atmospheric conditions.

To investigate the robustness of the as-synthesized ZIF-8@Luc, several techniques such as pH evaluation, temperature inactivation, presence of organic solvent, and evaporation/redispersion process were used (Figure 4). Regarding the pH influence, three conditions were tested using 50 mM Tris–HCl buffered at pH 5.0, 8.0, and 10.0 (Figure 4A). pH 8.0 was the optimum value for preserving the catalytic activity of the luciferase.^36^ ZIF-8 was able to preserve luciferase activity at pH 5.0 (remaining activity 50–60%); meanwhile, free luciferase was completely denatured (negligible BL signal). At this pH, mimicking the internal environment of tumor cells, the result suggests a higher resistance to conformational changes under acidic conditions when ZIF-8 is present, maybe due to the lack of mobility when luciferase is attached, thus causing protection of actives sites. To further confirm this, ZIF-8 was incubated in a solution of 50 mM Tris–HCl (pH ∼ 5) for 48 h. FT-IR was measured (Figure S9) and no signal was observed, indicating the collapse of ZIF-8; meanwhile, the pH was increased from 4.89 to 7.10. This fact explains the integrity protection of the luciferase since ZIF-8 could act as a sacrificial material to increase the pH value. At pH 10.0, different kinetic behavior of ZIF-8@Luc was observed including a significant BL signal decrease (∼95% at 200 s). It has been reported that enzymes attached to positively charged supports, such as ZIF-8 at pH 7–8, present stronger activity at lower pH values.^30,31^ On the other hand, when the heating process was carried out up to 50 °C, the BL signal of ZIF-8@Luc was reduced (80% signal loss) more than that of the free luciferase signal (40% signal loss), despite the thermal stability of ZIF-8. This could be due to the agglomeration of ZIF-8 occurring when the biocomposite is heated, thus leading to a decrease in the available catalytic sites of luciferase. Furthermore, luciferase stabilization, particularly at high temperatures, is enhanced with covalent attachments,^30,47^ this interaction being a minor contributor in our approach. In addition, other denaturing conditions were applied to ZIF-8@Luc including the presence of organic solvents (Figure 4C) and solvent evaporation (Figure 4D). The effect of several organic solvents on the ZIF-8@Luc dispersion was also explored.

We tested acetone, acetonitrile, ethanol, and isopropanol in a ratio of 1:1 v/v. As shown in Figure 4C, the presence of ZIF-8 allows the protection of luciferase against acetone (no significant BL signal loss) and ethanol (remaining BL signal of 25%), maybe due to its good dispersibility in organic solvents. The free luciferase lost its activity in the presence of all of the solvents with a decreased BL signal from 93 to 100%. Taking into account the polarities of organic solvents (acetone < ACN < 2-IPA < EtOH), ZIF-8, which is hydrophobic, will be stable/comfortable in this media and could protect the enzyme integrity better than that in other cases.

Regarding the evaporation/redispersion of ZIF-8@Luc, complete removal of the supernatant, after centrifugation, with a drying process overnight at 25 °C was performed. After redispersion, the BL signal was 7–12% compared to that of fresh ZIF-8@Luc (Figure 4D). No BL signal was observed after evaporation/redispersion of free luciferase in the same conditions.

### Characterization of ZIF-8@Luc and Michaelis–Menten Kinetics

Recyclability is one of the most important and desired features when enzymes are used due to the reduced production costs. This parameter (Figure S10) was assessed for ZIF-8@Luc by performing the reaction several times after the washing step (further information can be found in the Materials and Methods section). As observed, although the kinetic behavior slightly changed after the first use, the biocomposite could be used at least three times with a decent BL emission (100, 85, and 61% of the initial signal at 100 s, respectively). Despite the signal decrease, due to biocomposite loss in centrifugation/redispersion steps, this is an important achievement since free luciferase in the solution cannot be recycled, and, to the best of our knowledge, it has not been yet studied in other reported biocomposites.

**Figure 5:**
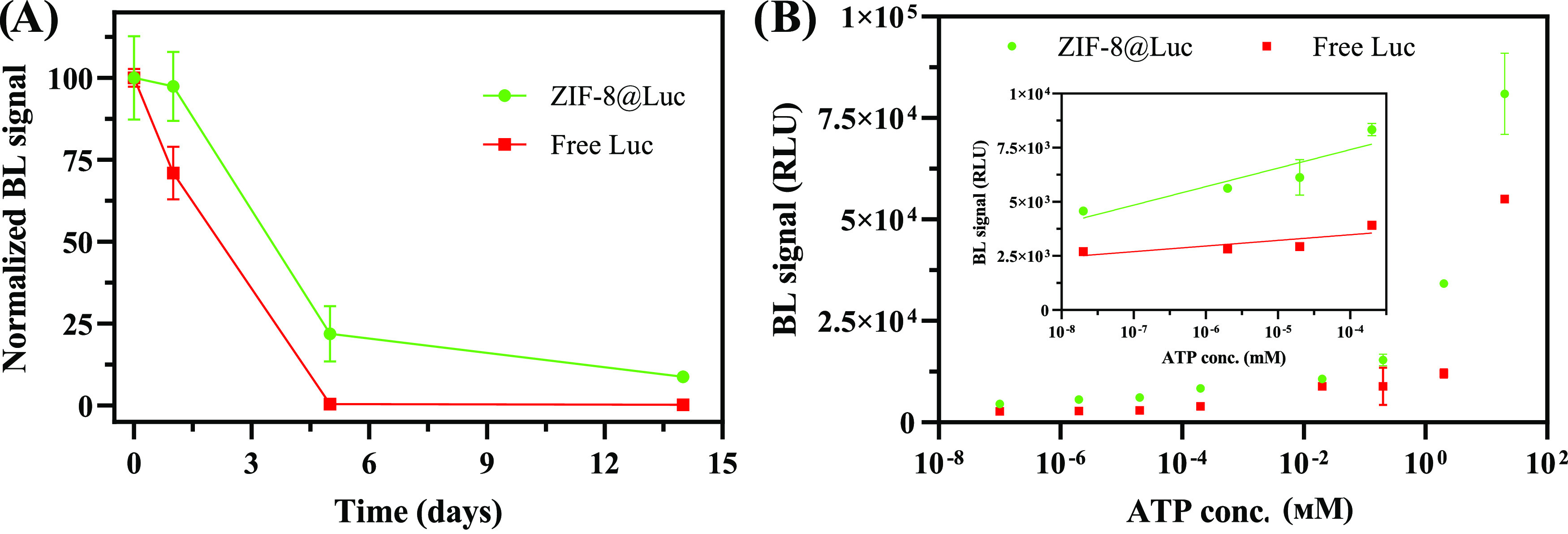
(A) Storage stability of ZIF-8@Luc at room temperature (25 °C). (B) Dose–response curve for ATP. BL signal at 20 min was used for calculating the average of four different replicates. The inset represents the linear correlation in the lower range of the BL signal vs log (ATP conc.).

To investigate medium long-term stability, two studies were performed in parallel at 4 and 25 °C, after the ZIF-8@Luc syntheses. BL signal was not detected for the free luciferase with storage time higher than 1 day at 25 °C, while ZIF-8@Luc was able to retain luciferase enzymatic activity (22 and 9%) up to 5 and 14 days after its synthesis, respectively (Figure 5A). The explanation of these results can be easily interpreted by p-XRD and FT-IR results (Figures 2B and S11). The BL loss is related to the progressive degradation of the ZIF-8 structure and subsequently the release of the enzyme into the medium, which remains unprotected. Furthermore, it can be hypothesized that the leaching of Zn(II) ions in the solution can decrease luciferase viability, as previously reported.^48^ In fact, MOFs are not stable in most of the buffers due to the interactions between metal and organic compounds, and ionic strength^49^ being Tris–HCl one of the most suitable buffer.^44^ On the contrary, luciferase is not stable in nonbuffered solutions. In this sense, the selection of the storage buffer for ZIF-8@Luc is a compromise and an optimal solvent cannot be identified. Future work will entail other alternatives such as the addition of stabilizers. A gradual decrease in BL signal during a 1 month period was observed at 4 °C (Figure S12) with about 20–25% remaining activity. In this case, the degradation of ZIF-8 in Tris–HCl was lower since the temperature was set at 4 °C. Additionally, free luciferase was incubated for 14 days at 4 °C and the comparison with the homologue biocomposite is shown in Figure S12. The free luciferase BL behavior is similar to that of the biocomposite, but the signal decays at a higher rate over time, highlighting the role of ZIF-8 in the stabilization of the BL signal leading to increased light output. Finally, the *K*_m_ for both ATP and d-LH_2_ were estimated. The results are summarized in Table 1. Although the *K*_m_ for ATP in the case of ZIF-8@Luc increased twice compared to that of free luciferase, there were no significant differences between the data considering the standard deviation. The small variation could be explained by diffusion barriers, a decrease in active sites, and conformational enzyme changes. Differently from previous literature, which reported ZIF-8 degradability in the presence of ATP,^50,51^ in this work, ATP is a cofactor required for luciferase catalytic activity; therefore, it is rapidly used by the enzyme luciferase in the first catalytic step of the bioluminescent reaction (adenylation of d-luciferin). In addition, since the luciferase is not encapsulated but rather immobilized onto ZIF-8, the accessibility of ZIF-8 to ATP is also limited. The precision in ATP detection studies was also investigated to assess the robustness of the results. The findings are summarized in Table S2. In all cases (intra- and interbatch), the RSD was ≤13%, even when different ATP concentrations and acquisition times were selected.

**Table 1:** Michaelis–Menten Kinetics for the Determination of *K*_m_^a^

	Michaelis–Menten constant, *K*_m_	
	ATP (μM)	d-LH_2_ (μM)
free luciferase	21 ± 5	50 ± 13
ZIF-8@Luc	49 ± 12	44 ± 11

a SD, *n* = 3 different batches, per duplicate each measure.

### Proof of Principle: ATP Quantification

The suitability of ZIF-8@Luc to quantify ATP was assessed. The dose–response curves, shown in Figure 5B, were obtained with the optimized conditions (see the Materials and Methods section). LODs for ATP were 2 × 10^–16^ and 2 × 10^–10^ mol for ZIF-8@Luc and free luciferase, respectively. This significant enhancement is due to the stabilization of the signal in the presence of MOF and confirms the suitability of this immobilization strategy.

### Comparison with Other Reported Methods

As far as we know, this is the first report exploring the immobilization of luciferase onto ZIF-8. However, other materials have been previously reported as carriers including magnetic nanoparticles (m-NPs),^52^ MOFs,^30,31^ graphite,^18^ silica,^21^ and other nanomaterials^17,19^ to enhance the luciferase kinetic response and its stability properties. Table S3 summarizes some important features from the most recent contributions (over the last 20 years) in comparison to our work. Although several host materials have been used with different chemical behaviors, an exhaustive characterization of the resulting biocomposite is generally lacking, together with cost-effectiveness and sustainability analysis. Regarding the LODs, very few works have reported values comparable to that described by Cruz-Aguado et al.^21^ and slightly higher than the values reported by Wang and collaborators.^19^ On the other hand, the *K*_m_ values for ATP are higher in some cases,^17,52^ similar when other MOF materials are used^30,31^ and for sugar-modified sol–gel silica^21^ was 10-fold lower, suggesting different accessibility of the luciferase for the ATP substrates. The synthesis times were in the same order for all procedures^17,18,30,31,52^ except for sugar-modified sol–gel silica^21^ and nanofiber membranes,^19^ which were notably higher. Despite the importance of reusability and storage stability, there are no reports about these values (except from Cruz-Aguado’s group^21^). No information was provided about the synthesis process reproducibility, protection of luciferase against organic solvents or processes such as evaporation/redispersion of freezing/thawing, all these aspects studied in the present work. Considering the above-mentioned features as well as the simplicity and straightforward synthesis of our approach, this method can be considered an attractive and highly recommended procedure to immobilize not only luciferases but also other enzymes to enhance their properties.

## Conclusions

For the first time, we investigated the immobilization of luciferase and ZIF-8. We have optimized the synthesis and characterized the resulting biocomposite. As confirmed by BL emission and kinetic measurements, the presence of MOF did not affect the catalytic activity of the enzyme, providing instead a 40% signal enhancement. Furthermore, the conformation that the protein acquires after the attachment together with the host environment of ZIF-8 leads to an increase in thermal resistance at room (∼25 °C) and low temperatures (−20 °C) and better chemical stability at acidic pH (pH ∼ 5.0) and in the presence of organic solvents (acetone and ethanol) compared to free luciferase (remaining activity from 25 to 95%). Despite these promising findings, further studies will be required to achieve adequate stabilization at alkaline pH and at higher temperatures (50 °C) by exploring changes in the MOF structure (for instance, by adding functionalities). Reusability studies were performed and ZIF-8@Luc could be used at least three times with decent BL response (80% at 80 s), leading to a significant assay cost reduction. The achieved LOD (0.2 fmol) for ATP prompts future applicability of ZIF-8@Luc for ATP sensing, also in portable analytical devices. The present method could thus represent a synergic strategy, together with mutagenesis of the protein, to enhance BL performance and stability and could serve as a guide, after full characterization, for other researchers to use MOFs as host materials for proteins.

## Supporting Information Available

Reagents and materials, instrumentation, solution preparations, MOF syntheses, and bioluminescent emission kinetics of luciferase/MOFs; bioluminescent emission kinetics of different biocomposites; IR spectra of ZIF-8; optimization of biocomposite ZIF-8@Luc synthesis conditions: stirring type, amount of ZIF-8, and orbital stirring time; washing and reusability studies; and storage, precision results, and table of comparison of ZIF-8@Luc with other biocomposites (PDF)
