# Supplementary material for: Novel Nanozeolitic Imidazolate Framework (ZIF-8)–Luciferase Biocomposite for Nanosensing Applications
Source: Anal Chem. 2022 Dec 6;95(4):2540–7. doi: 10.1021/acs.analchem.2c05001 (PMC9893222; doi:10.1021/acs.analchem.2c05001)
Supplement: Supplementary file 1 — ac2c05001_si_001.pdf [file ac2c05001_si_001.pdf]

Supporting information

A novel nano zeolitic imidazolate framework (ZIF-8) – luciferase biocomposite for nanosensing applications

Hector Martínez-Pérez-Cejuela[†,‡], Denise Gregucci[†,¥], Maria Maddalena Calabretta[†,¥], Ernesto Francisco Simó-Alfonso[‡], José Manuel Herrero-Martínez[‡] and Elisa Michelini[†,¥,□]*

[†]Department of Chemistry "Giacomo Ciamician", University of Bologna, Via Selmi 2, 40126, Bologna, Italy

[‡]Department of Analytical Chemistry, University of Valencia, C/Dr. Moliner, 50, 46100-Burjassot, Valencia, Spain

[¥]Center for Applied Biomedical Research (CRBA), Azienda Ospedaliero-Universitaria Policlinico S. Orsola-Malpighi, 40138, Bologna, Italy

[□]Health Sciences and Technologies Interdepartmental Center for Industrial Research (HSTICIR), University of Bologna, 40126, Bologna, Italy

*Prof. Dr. Elisa Michelini: elisa.michelini8@unibo.it

Table of contents

Page S3. Reagents and materials

Pages S3-S4. Solution preparations

Pages S4. Instrumentation

Pages S4-S6. MOF syntheses

Pages S7. p-XRD from pure MOFs (Figure S1)

Pages S8. FT-IR from pure MOFs (Figure S2)

Pages S9. Surface area from pure MOFs (Table S1)

Pages S10. Bioluminescent emission kinetics of luciferase/ZIF-8, luciferase/MIL-101(Al) and luciferase/UiO-66 dispersions (Figure S3)

Page S11. Bioluminescent emission kinetics of ZIF-8@Luc, MIL-101(Al)@Luc and UiO-66@Luc biocomposites (Figure S4)

Page S12. FT-IR spectra of free luciferase, ZIF-8 and biocomposite and Tris-HCl (Figure S5)

Pages S13-S14. Results discussion from synthesis optimization

Page S14. Optimization of biocomposite synthesis, stirring conditions (Figure S6)

Page S15. Optimization of biocomposite ZIF-8@Luc synthesis conditions, amount of ZIF-8 and orbital stirring time (Figure S7)

Page S16. ZIF-8@Luc washing study (Figure S8)

Page S16. FT-IR spectra of ZIF-8 in contact with acidic conditions (Figure 9)

Page S17. Reusability of ZIF-8@Luc (Figure S10)

Page S17. FT-IR spectra of ZIF-8 in contact with Tris-HCl (Figure S11)

Page S18. Storage study of ZIF-8@Luc (Figure S12)

Page S19. Precision study of ZIF-8@Luc BL response (Table S2)

Page S20. Comparison between ZIF-8@Luc performance and other reported biocomposites (Table S3)

Pages S21-S22. References

MATERIALS AND METHODS

Reagents and materials. zinc nitrate hexahydrate, cobalt (II) chloride, aluminum chloride hexahydrate, zirconium (IV) chloride, HMIM, terephthalic acid (TA), n-butylamine were obtained from Sigma-Aldrich (Milwaukee, WI, USA). Aqueous dispersion of AuNP stabilized with sodium citrate (average diameter size 20 nm, $6.54 \cdot 10^{11}$ particles mL^{-1}) were obtained from Alfa Aesar. All the solvents (e.g. acetonitrile, isopropanol, ethanol, acetone, methanol (MeOH), dimethyl formamide (DMF) and others) were HPLC grade and provided from VWR International Eurolab (Milan, Italy). Trizma® hydrochloride, sodium phosphate monobasic, sodium chloride, imidazole, ATP (disodium salt hydrate), citric acid, sodium citrate and magnesium chloride hexahydrate BioXtra were from Sigma-Aldrich. Beetle luciferin potassium salt (D-LH_2) was from Promega (Madison, WI, USA).

All other reagents used were of analytical grade unless otherwise stated. MilliQ (MQ) water was purified in Rephile Bioscience Millipore (reference A+).

Solution preparations. Lew buffer was prepared at the final concentration of 50 mᴍ of sodium phosphate, 300 mᴍ of sodium chloride and 250 mᴍ of imidazole (final volume 50 mL). Then, the pH was adjusted to 8.0 with NaOH 1 ᴍ. Tris-HCl buffer was prepared at 50 mᴍ and the pH was adjusted to 7.8 with NaOH 1 ᴍ. Both buffers were kept at 4ºC for their use. Citric/citrate buffer was prepared mixing 0.1 ᴍ individual solutions (35/65 v/v, respectively) and the pH was adjusted to 5.5 with NaOH 0.5 ᴍ. Magnesium chloride solution was prepared weighing 20 mg and solving the salt in 10 mL of MQ water. The solution was kept at room temperature for further use.

ATP stock solution (20 mᴍ) was monthly prepared dissolving the appropriate amount (22 mg) in 2 mL of Tris-HCl buffer (50 mᴍ, pH 7.8). After the homogenization, the total volume was divided into 4 Eppendorf tubes. All were protected from light with

aluminium paper and were kept at -20 ºC for their use. Working standard solutions (2 mM) were daily prepared by proper dilution with the same buffer and kept at 4 ºC.

Luciferin solution (1 mM) was prepared by dissolving 8 mg of luciferin potassium salt with 25 mL of citric/citrate buffer 0.1 M and stirred in an ice batch for 20 min in the darkness. Then, the volume was adjusted to 25 mL and divided in 5 mL-fractions in different 15 mL Falcon tubes. All were protected from light with aluminium paper and kept at -20 ºC, except the working fraction that was placed at 4ºC for a week.

Instrumentation. Incu·ShakerTM model 10LR (Benchmark, Sayreville NJ USA) was used to perform the synthesis of the biocomposite. Varioskan LUX (Thermo Scientific, Waltham, MA, USA) with automatic injection was used to perform all the BL assays. For the characterization of the ZIF-8: micrographs were taken with a transmission electron microscopy (TEM) model JEM-1010 JEOL coupled to AMT RX80 digital camera (Akishima, Japan). Also, scanning electron microscopy (SEM) images were acquired with a SEM microscope model Hitachi S-4800 (Ibaraki, Japan) coupled to a retro dispersive electron detector. Analysis with Powder X-ray Diffraction (p-XRD) was performed to register the spectra of the materials using a D8 Advance A25 diffractometer (Bruker, Hamburg, Germany). Also, attenuated total reflection Fourier-transform infrared (FT-IR) spectra of ZIF-8 materials were acquired with the equipment Bruker FT-IR spectrometer (Bremen, Germany) model Tensor 27 using a nine reflection diamond/ZnSe DuraDisk plate. Adsorption-desorption nitrogen isotherms were performed using S5 Micromeritics ASAP2020 instrument (Norcross, USA) at 77 K and the specific surface area was obtained with the mathematic model Brauner-Emmet-Teller (BET). For the measurements of all MOFs, the samples were previously degassed at 425 K using a heating rate of 5 K min^{-1} for 5 h.

MOF syntheses. *MIL-101(Al)* was synthesized according to our previous work[1] with slight modifications. Briefly, 2 mmoles of Al (III) chloride hexahydrate and 3 mmoles of TA acid were mixed in 40 mL of DMF. The mixture was homogenised in the ultrasonic bath. Then, the solution was placed into a Teflon-lined reactor in different 6 mL-vials and the reaction was performed for 72 h setting the temperature constant at 130 °C. After this period, resulting yellow solid was cooled at room temperature and collected by centrifugation (18,000 g) for 10 min and washed three times with DMF (15 mL) and one with ethanol (15 mL). Finally, the MOF was placed in an oven overnight at 100 ºC.

UiO-66 was synthesized following a procedure described elsewhere[2] with minor changes. An initial mixture containing 1 mmol of zirconium tetrachloride and 1 mmol of TA in DMF (final volume 50 mL) was done. After the homogenization with vortex and ultrasonic bath, the mixture was placed into a Teflon-lined reactor in different 6 mL-vials for 48 h at 120 ºC. After the synthesis time, the yellowish solid was cooled at room temperature and collected by centrifugation (18,000 g) for 10 min. Then, the MOF was washed twice with DMF (25 mL) and redispersed in 50 mL of MeOH under stirring for 12 h. The as-synthesized solid was dried in the oven at 100 ºC overnight.

ZIF-8 was synthesized according to our previous work.[3] ZIF-8 was prepared mixing under vigorously stirring a solution (i) of 100 mL of MeOH containing 39.52 mmoles of HMIM into a solution (ii) of 100 mL of MeOH containing 4.94 mmoles of zinc nitrate hexahydrate. A white dispersion was formed in the first 10 min of the reaction, and the dispersion was aged for 24 h at room temperature under stirring. The separation was achieved by centrifugation at 18,000 g for 10 min. Then, the white MOF was rinsed with 25 mL MeOH (x 3) and dried at 50°C overnight. The MOF was grinded and sieved collecting the fraction in the range $100 \leq x \leq 200$ µm in order to improve the reproducibility of the re-sults.

NH_2-ZIF-8 and its composite *AuNP@NH_2-ZIF-8* were synthesized according to our previous work[4]. For the NH_2-ZIF-8 synthesis, a solution (i) of the HMIM and butylamine (9.87 mmoles of each one) was prepared in 50 mL MeOH and slowly poured under stirring into a 50 mL of methanolic solution (ii) containing 2.47 mmoles of zinc nitrate. A white dispersion was instantly formed, and the reaction was carried out under gentle stirring at room temperature for 24 h. The solid was separated by centrifugation at 18,000 g for 10 min and washed with MeOH (25 mL) per triplicate and dried in an oven at 50ºC for 8 h. For the attachment of AuNPs to the NH_2-ZIF-8 network: 250 mg of dried and sieved MOF ($100 \leq x \leq 200$ μm) were weighed in a 15 mL-Falcon tube. Then, 10 mL of commercial AuNPs dispersion were poured and the mixture was magnetically stirred for 10 min. After the separation of the dispersion by filtering with a nylon membrane, the solid had acquired a pale pink tonality. The pinkish MOF was rinsed with 5 mL of citrate buffer (20 mM at pH 6.0) twice. The gold attachment was performed three times when the saturation of the sorbent was evidenced by UV–Vis spectra of the supernatants. Finally, the as-synthesized AuNP@NH_2-ZIF-8 was washed with 10 mL of MeOH per duplicate and dried overnight at 50 ºC.

ZIF-67 was synthesized as reported elsewhere[5] with minor modifications. For instance, two solutions were prepared: i) 0.77 mmoles of Co(II) precursor was weighed and solved in 1.5 mL of MQ water; and ii) 33.5 mmoles of HMIM were solved in 10 mL of MQ water. The former solution was added dropwise under vigorous stirring (not controlled) for 5 min. An intense purple colour was instantly observed after the addition of the first drop- Then, the reaction was kept under stirring for 8 h at atmospheric conditions. After this period, the solid was collected by centrifugation (10 min, 18,000 g) and washed twice with water (5 mL). The resulting solid was dried in an oven (80 ºC) for 12 h.

All the MOF materials were grinded and sieved collecting the fraction in the range $100 \leq x \leq 200$ μm in order to improve the reproducibility of the results.

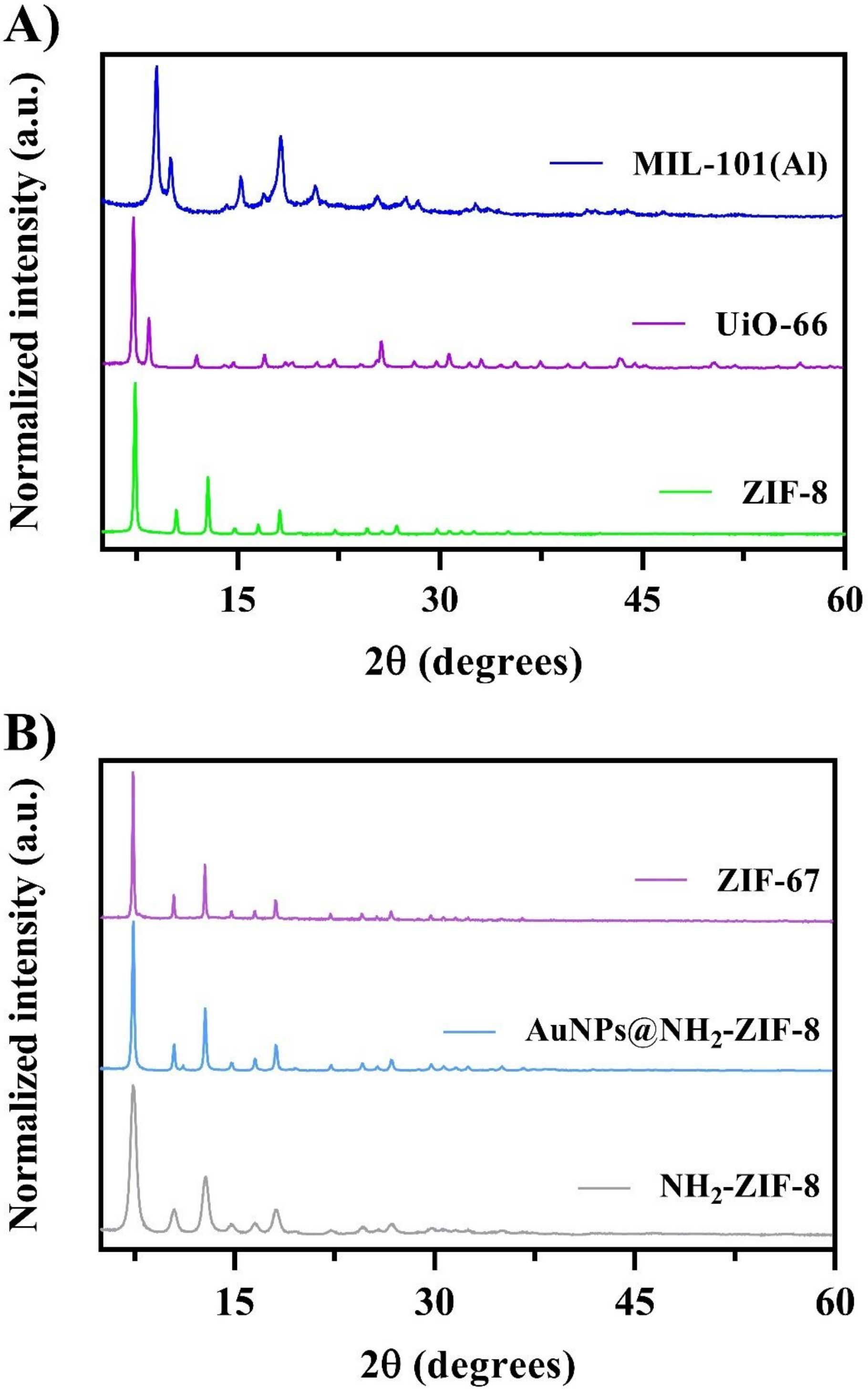

Figure S1. p-XRD spectra from A) the dispersion study: UiO-66, MIL-101 (Al) and ZIF-8; and B) ZIF-n family used in this work (from 2ϴ = 5-60).

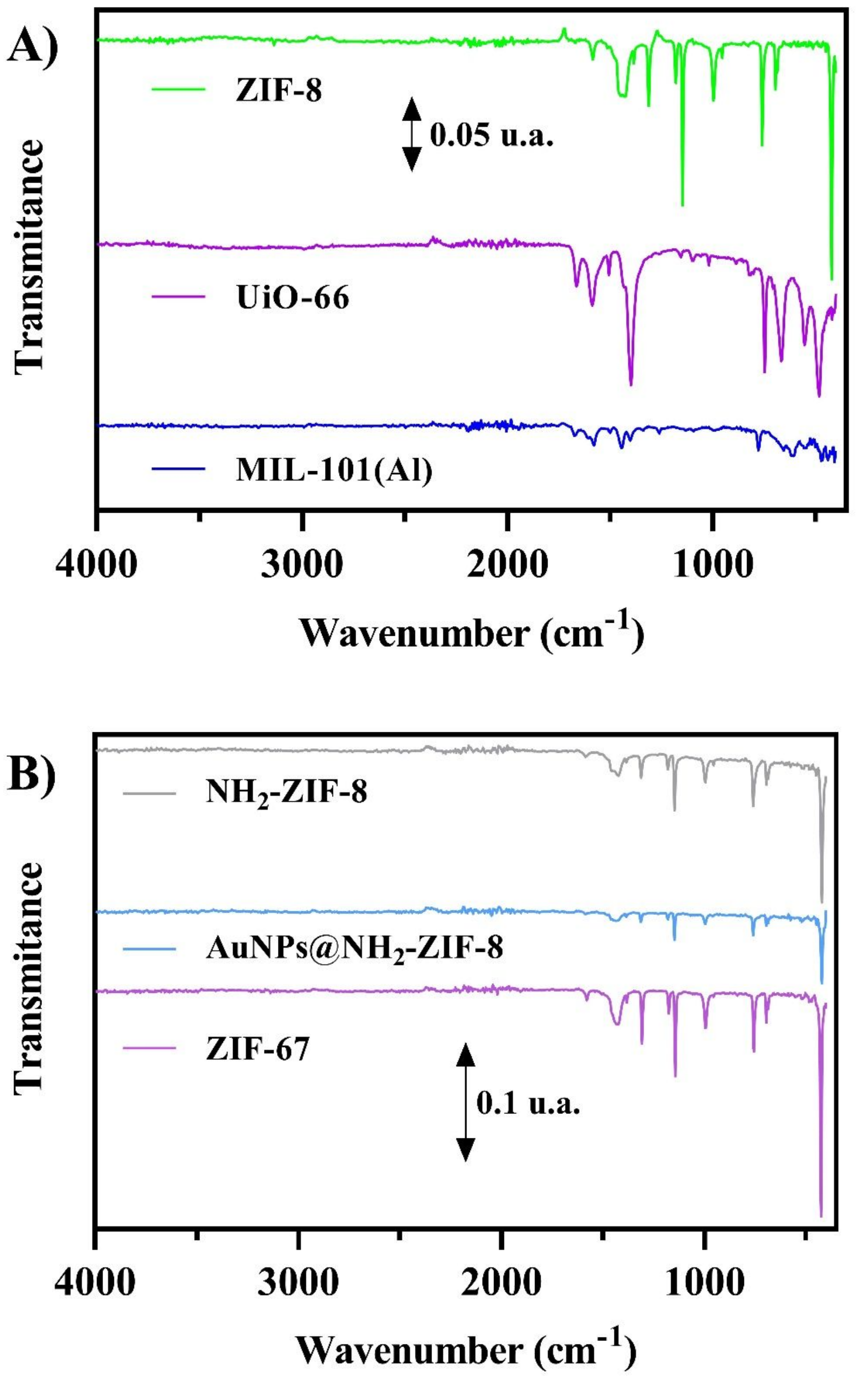

Figure S2. FT-IR spectra from A) the dispersion study: UiO-66, MIL-101 (Al) and ZIF-8; and B) ZIF-n family used in this work (350-4000 cm^{-1}).

Table S1. Surface areas from all MOF used in this work.

MOF material	Surface area (m^2 g^{-1})
MIL-101 (Al)[1]	3038
UiO-66[1]	810
ZIF-8[1]	1456
NH_2-ZIF-8[1]	1365
AuNP@NH_2-ZIF-8[1]	1167
ZIF-67[2]	316

[1]Obtained with ASAP 2020 analyzer; Experimental conditions are written in the Instrumentation Section; [2]Obtained from the bibliography (ref. 5)

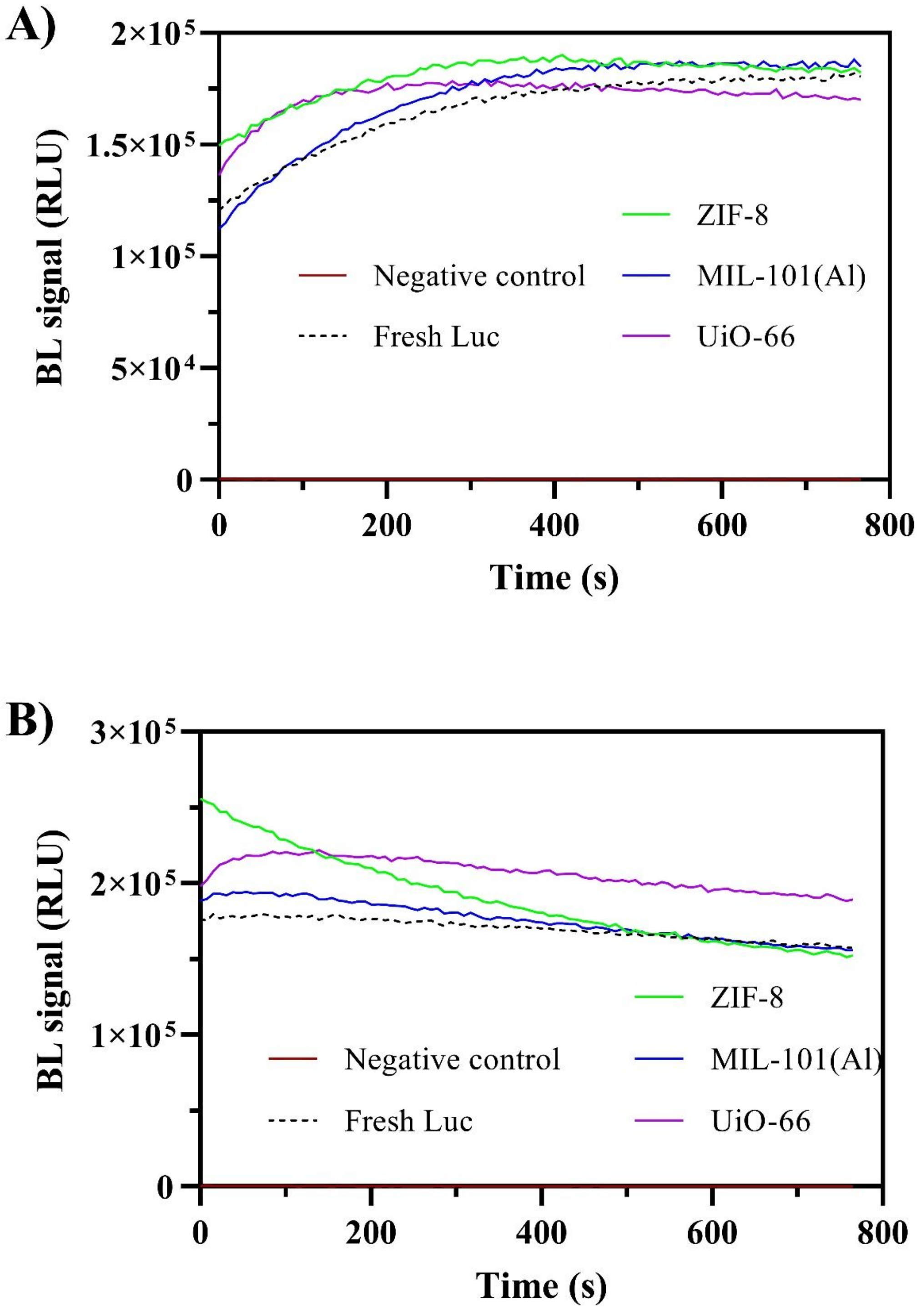

Figure S3. Bioluminescent emission kinetics of luciferase/ZIF-8, luciferase/MIL-101(Al) and luciferase/UiO-66 dispersions. Final concentration of each MOF: A) 40 mg L^{-1}; and B) 80 mg L^{-1}. More details can be found in Materials and Methods.

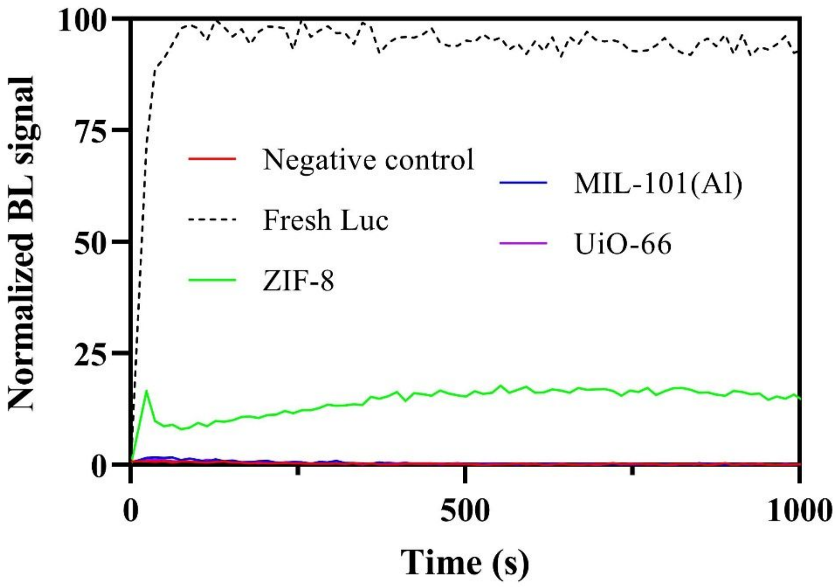

Figure S4. Bioluminescent emission kinetics of ZIF-8@Luc, MIL-101(Al)@Luc and UiO-66@Luc biocomposites. Experimental conditions: synthesis with magnetic stirring for 1 h at 4 ºC; 0.5 mg MOF; 0.1 mg mL^{-1} luciferase in 200 µL of 50 mм Tris-HCl pH 7.8. More details can be found in Materials and Methods.

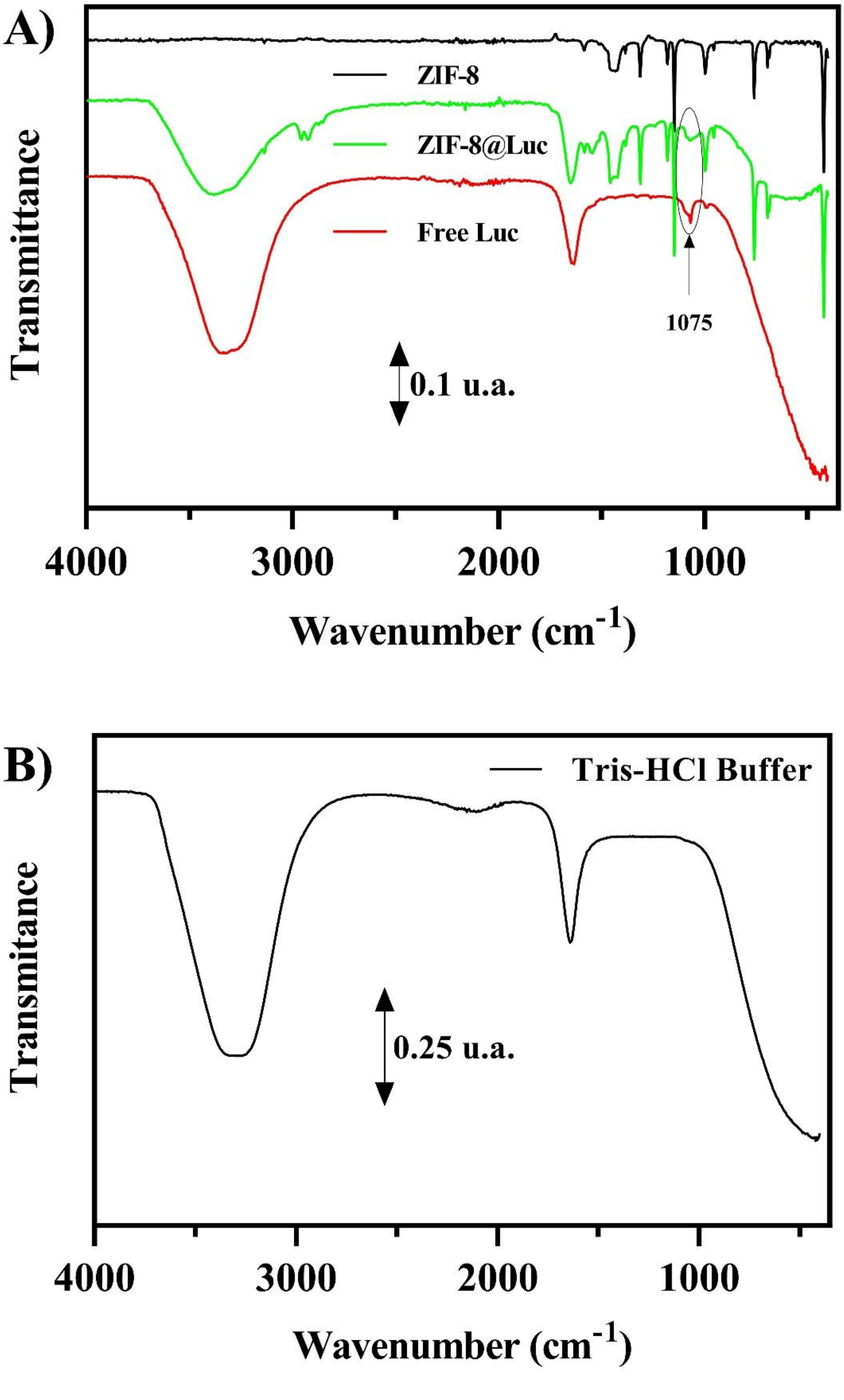

Figure S5. FT-IR spectra from 4000 to 400 cm^{-1} of A) free luciferase (0.1 mg mL^{-1}), pure ZIF-8 (powder) and biocomposite (50 mg mL^{-1}) and B) 50 mM Tris-HCl pH 7.8.

RESULTS AND DISCUSSION

Bearing in mind the decrease of BL signal (~80%) from ZIF-8@Luc compared to fresh luciferase in the preliminary studies (results from **Figure S4**), the first study was focused on the stirring source. As stated in "Rational and design of the luciferase-MOF biocomposite" (previous section), the importance of mild conditions for protecting enzyme functionally is crucial to achieve the final biocomposite. For this purpose, different syntheses were performed using magnetic, orbital and vortex-assisted stirring sources. As it can be observed in **Figure S6**, the highest BL signal was achieved when orbital stirring was used in which a ~ 15% in BL signal decrease was found than the fresh luciferase, meanwhile the lowest was observed with magnetic stirring (~ 73% in BL signal decrease). Furthermore, around 5% of free luciferase (data not shown) was quantified in the supernatant when vortex-assisted synthesis was studied. To further confirm the best synthesis route, the free luciferase was treated with the same procedure (named as control, see inset **Figure S6**) of ZIF-8@Luc synthesis and the use of orbital stirring was thus selected for subsequent studies. Unexpectedly, the ZIF-8 could stabilize the luciferase structure, even when magnetic stirring was tested (**Figure S6**), thus confirming its protective role on enzyme catalytic activity.

Next, the amount of ZIF-8 and time of synthesis were optimized. In the case of ZIF-8 amount, the studied range was from 0.25 to 0.75 mg for 1 h of synthesis. The results are depicted in **Figure S7A**. The best amount of ZIF-8 was 0.25 mg, which enabled to obtain the highest BL intensity. Comparing the BL signals, a BL signal decrease of 82% and 87% was observed using 0.50 and 0.75 mg of ZIF-8, respectively. Most presumably, higher ZIF-8 amounts form aggregates due to its hydrophobicity and, subsequently, reduce the number of available active sites for enzyme embedding.

We also investigated the synthesis time in the range 15-60 min (**Figure S7B**) using 0.25 mg of ZIF-8. As observed, there is no significant difference between 30-60 min of synthesis time. However, both conditions were slightly better than 15 min, probably because the equilibria time was not achieved in 15min. Therefore 30 min was the selected time to investigate the ZIF-8@Luc performance.

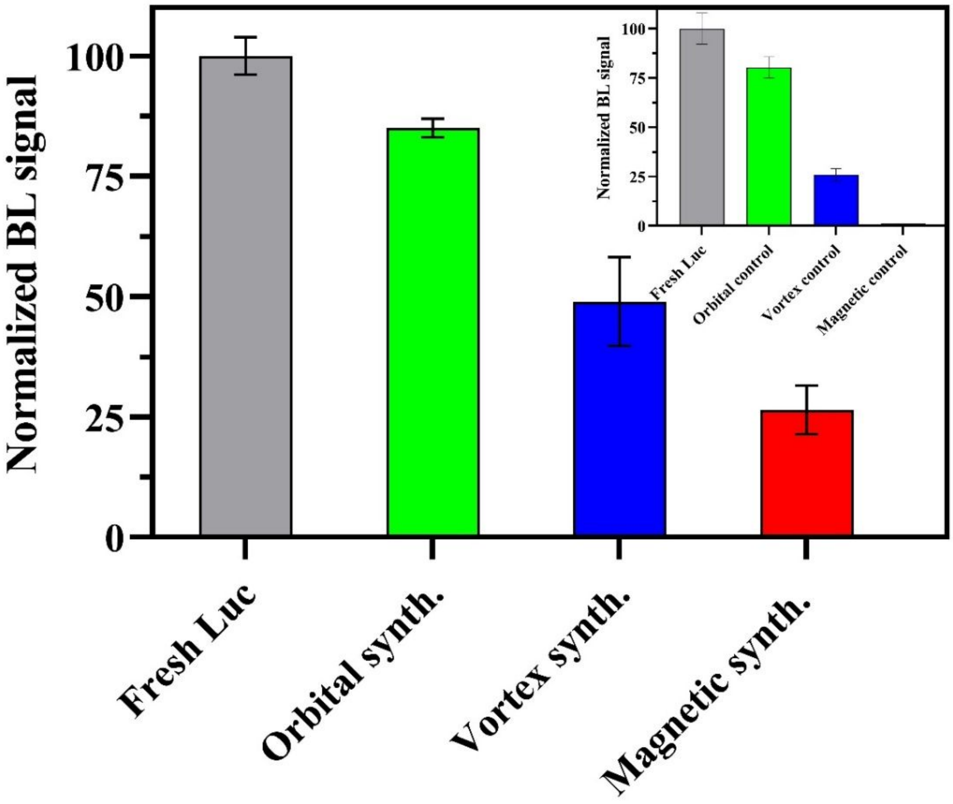

Figure S6. Optimization of biocomposite synthesis. Bioluminescent signal of the ZIF-8@Luc obtained with orbital, vortex or magnetic stirring. Inset shows the results from the same procedure using only free luciferase without ZIF-8. Experimental conditions: synthesis for 1 h at 4 ºC; 0.1 mg mL^{-1} luciferase in 200 µL of 50 mM Tris-HCl pH 7.8. More details can be found in Materials and Methods.

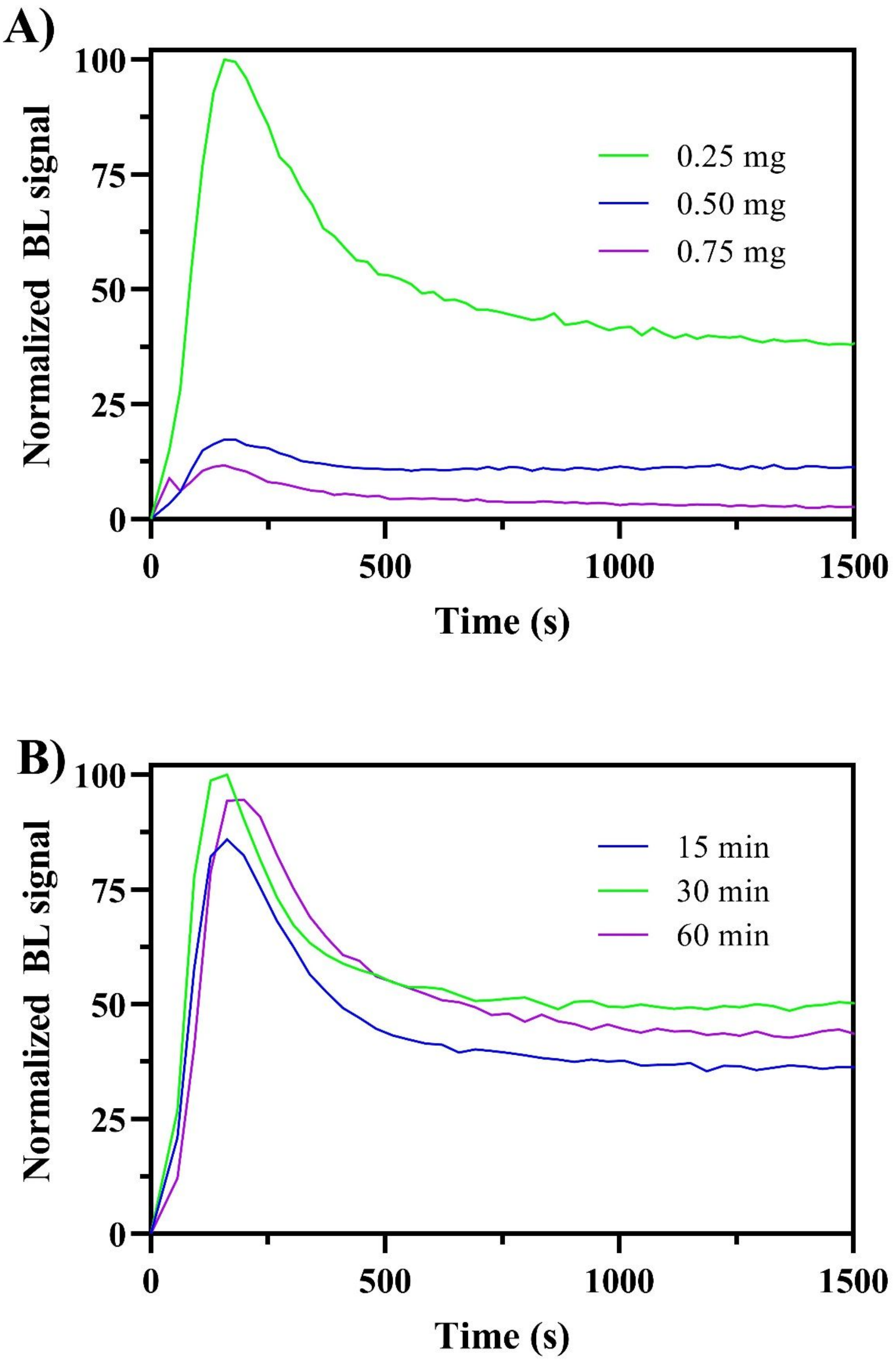

Figure S7. Optimization of biocomposite synthesis conditions. Synthesis optimization studies including A) amount of ZIF-8; and B) orbital stirring time. Experimental conditions: 0.1 mg mL^{-1} luciferase in 100 µL of 50 mM Tris-HCl pH 7.8. More details can be found in Materials and Methods.

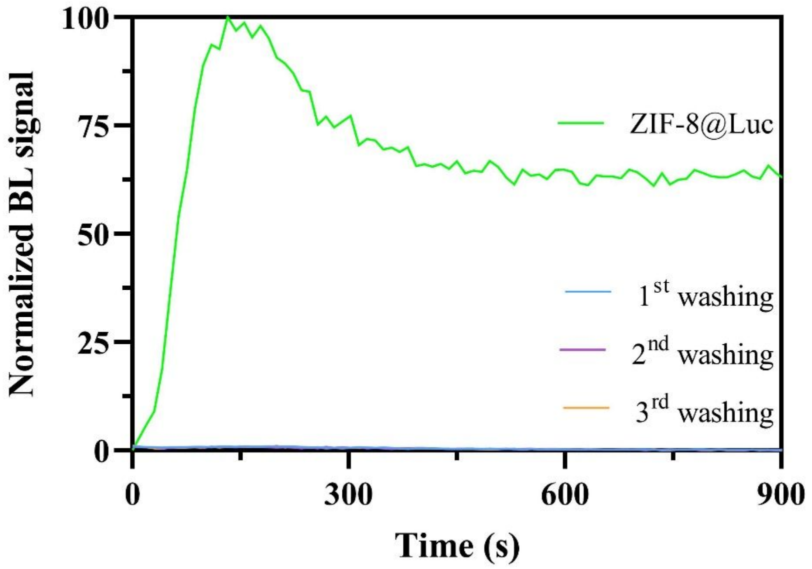

Figure S8. Study of luciferase leaking from the as-synthesized biocomposite after several washing steps with 50 µL of 50 mм Tris-HCl pH 7.8. Experimental conditions: orbital sitirring for 30 min at 4 ºC; 0.1 mg mL^{-1} luciferase in 100 µL of 50 mмTris-HCl pH 7.8. More details can be found in Materials and Methods.

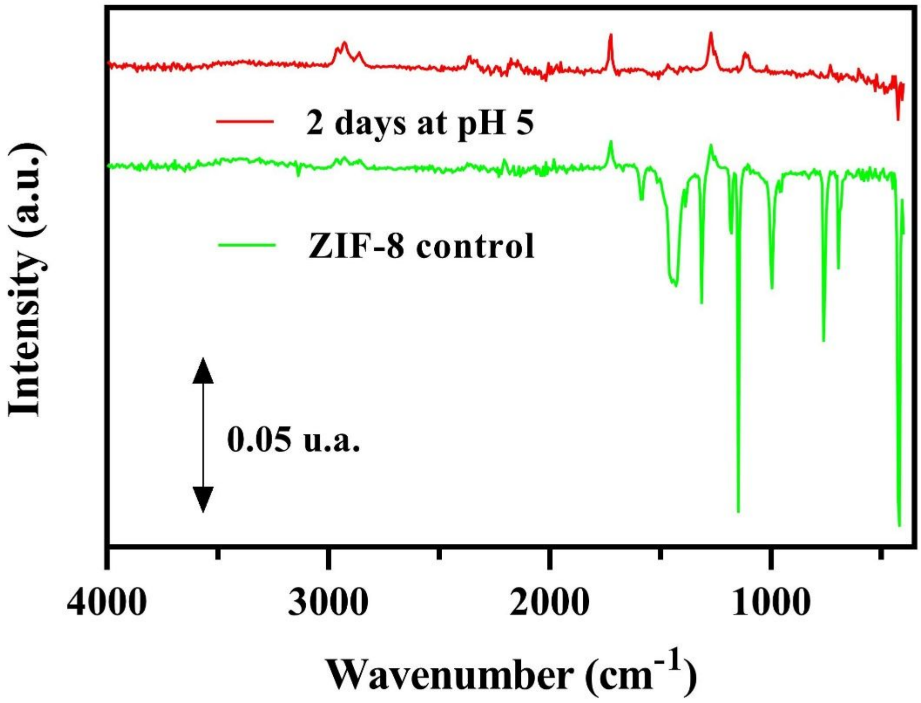

Figure S9. FT-IR spectra from 4000 to 400 cm^{-1}of ZIF-8 incubated for 2 days in 50 mM Tris-HCl at pH 5 (red line) and ZIF-8 control (green line)

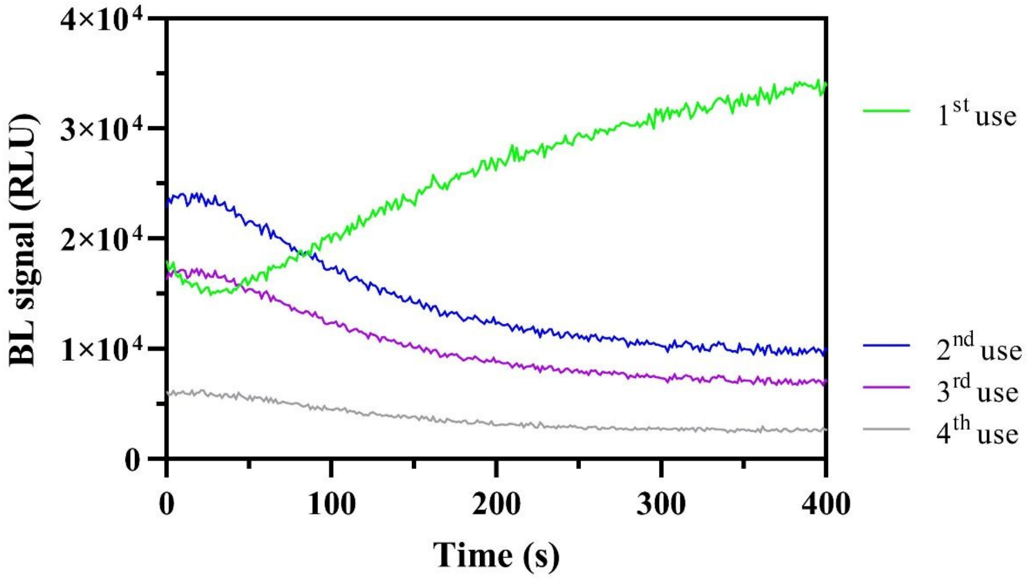

Figure S10. Reusability study of the ZIF-8@Luc after consecutive uses. Synthesis conditions orbital sitirring for 30 min at 4 ºC; 0.1 mg mL-1 luciferase in 100 µL of 50 mᴍTris-HCl pH 7.8. After the first use, the ZIF-8@Luc was washed twice with 50 µL of Tris-HCl (50 mᴍ, pH 7.8). The washed pellet was redispersed with 100 µL of Tris-HCl. More details can be found in Materials and Methods.

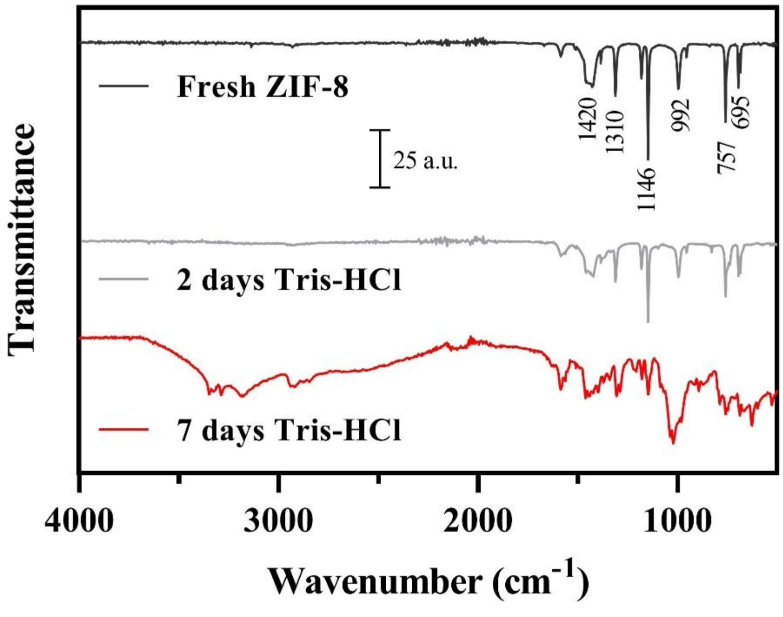

Figure S11. FT-IR spectra of ZIF-8 from 4000 to 500 cm^{-1}. Black line: freshly prepared without any further treatment; grey and red lines: ZIF-8 incubation with 50 mᴍ Tris-HCl pH 7.8 at room temperature for 2 and 7 days, respectively.

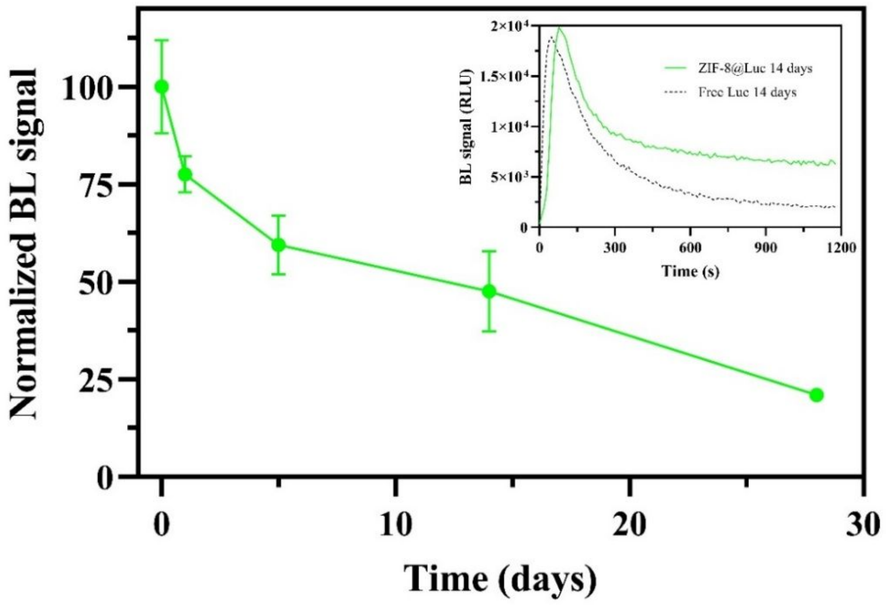

Figure S12. Storage stability study of ZIF-8@Luc over the time at 4ºC. Synthesis conditions: orbital sitirring for 30 min at 4 ºC; 0.1 mg mL^{-1} luciferase in 100 µL of 50 mᴍ Tris-HCl pH 7.8.. Inset represents the comparison of BL response between ZIF-8@Luc and free luciferase after incubation for 12 days at 4 ºC. More details can be found in Materials and Methods.

Table S2. Precision of ZIF-8@Luc BL response along different kinetics studies. Low, medium and high ATP concentrations were analysed at three different acquisition time (300, 600 and 1200 s).

**[ATP] mM**	**Time (s)**	**Precision (RSD, %)**	
		**Intra-batch[1]**	**Inter-batch[2]**
0.02	300	4.3	5.1
	600	6.4	9.6
	1200	8.3	7.8
0.2	300	3.7	12.7
	600	4.7	10.6
	1200	9.3	13.0
2	300	2	5.8
	600	4	3.7
	1200	1.2	5.0

[1]For intra-batch values, three different ZIF-8@Luc were synthesized in the same day using the same luciferase purification, MOF synthesis and reagent batch; [2]For inter-batch values, four different ZIF-8@Luc were synthesized along four different weeks using different luciferase purification, MOF synthesis and reagent batch.

Table S3. Comparison between optimized ZIF-8@Luc performance with other reported methods for immobilization of Luc onto functional materials in the past 20 years.

Luciferase specie	Carrier (amount/preparation time)	Luc attachment conditions	LODs (moles)	Km – ATP (μM)	Remarkable features	Ref.[1]
Photinus pyralis	Alkyl-substituted S4B (- / 1 h)	30 min at 4 ºC	-	150-1300	Slight enhancement of thermal stability	[17][6]
Luciola Mingrelica	Graphite (10x5x0.6 mm sections / -)	20 min at 2 ºC	-	-	Activity lost after 1000 s; Electric field causes irreversible damage to the enzyme (~75 %)	[18][7]
**-**	PVA-*co*-PE nFB mem. (foursquare film 1 cm side / 2.5 days)	24 h at 4 °C	$1.5 \cdot 10^{-14}$	-	Portable membrane	[19][8]
Photinus pyralis	Sugar-modified sol-gel silica (62 μL / 5 days)	5 days at 4 ºC	$2 \cdot 10^{-17}$	5	Reusability of 5 cycles along 1 month	[21][9]
Synthetic	NH_2-MIL-88 (Fe) - MOF (1 mg / 8 h)	60 min at 4 ºC	-	50	Increased thermal and chemical stability	[30][10]
Synthetic	MIL-53 (Al) - MOF (1 mg / 3 days)	60 min at 4 ºC	-	50	Enhanced thermal stability	[31][11]
Photinus pyralis	Ni(II)/Cu(II) m-NPs (2 mg / 3.5 days)	20 min at 4 ºC	-	300	Slight enhancement of thermal stability, poor reusability	[52][12]
Photinus pyralis mutant	ZIF-8 - MOF (0.25 mg / 1 day)	30 min at 4 ºC	$2 \cdot 10^{-16}$	49	Reusability: up to 3 times; RSD: ≤14 %; Storage: 14 d (50 % remaining activity); Organic solvent, acidic and freeze/thaw protection	This work

* **Abbreviations:** S4B: sepharose 4B; PVA-*co*-PE: poly(vinyl alcohol-co-ethylene); nFB mem: nanofibers membrane; m-NPs: magnetic nanoparticles; MIL: matériaux de l′institut Lavoisier; ZIF: zeolitic imidazolate framework; [1]Literature has also referred to the paper numbering to facilite the reading

REFERENCES

(1) Zatrochová, S.; Martínez-Pérez-Cejuela, H.; Catalá-Icardo, M.; Simó-Alfonso, E. F.; Lhotská, I.; Šatínský, D.; Herrero-Martínez, J. M. Development of Hybrid Monoliths Incorporating Metal–Organic Frameworks for Stir Bar Sorptive Extraction Coupled with Liquid Chromatography for Determination of Estrogen Endocrine Disruptors in Water and Human Urine Samples. *Microchim. Acta* **2022**, *189*, 92.

(2) Hu, Q.; Chen, Y.; Li, M.; Zhang, Y.; Wang, B.; Zhao, Y.; Xia, J.; Yin, S.; Li, H. Construction of NH2-UiO-66/BiOBr Composites with Boosted Photocatalytic Activity for the Removal of Contaminants. *Colloids Surfaces A Physicochem. Eng. Asp.* **2019**, *579*, 123625.

(3) Martínez-Pérez-Cejuela, H.; Mompó-Roselló, Ó.; Crespí-Sánchez, N.; Palomino Cabello, C.; Catalá-Icardo, M.; Simó-Alfonso, E. F.; Herrero-Martínez, J. M. Determination of Benzomercaptans in Environmental Complex Samples by Combining Zeolitic Imidazolate Framework-8-Based Solid-Phase Extraction and High-Performance Liquid Chromatography with UV Detection. *J. Chromatogr. A* **2020**, *1631*, 461580.

(4) Martínez-Pérez-Cejuela, H.; Pravcová, K.; Česlová, L.; Simó-Alfonso, E. F.; Herrero-Martínez, J. M. Zeolitic Imidazolate Framework-8 Decorated with Gold Nanoparticles for Solid-Phase Extraction of Neonicotinoids in Agricultural Samples. *Microchim. Acta* **2021**, *188*, 197.

(5) Qian, J.; Sun, F.; Qin, L. Hydrothermal Synthesis of Zeolitic Imidazolate Framework-67 (ZIF-67) Nanocrystals. *Mater. Lett.* **2012**, *82*, 220–223.

(6) Yousefi-Nejad, M.; Hosseinkhani, S.; Khajeh, K.; Ranjbar, B. Expression, Purification and Immobilization of Firefly Luciferase on Alkyl-Substituted Sepharose 4B. *Enzyme Microb. Technol.* **2007**, *40*, 740–746.

(7) Palomba, S.; Berovic, N.; Palmer, R. E. Bioluminescence of Monolayers of Firefly Luciferase Immobilized on Graphite. *Langmuir* **2006**, *22*, 5451–5454.

(8) Wang, W.; Zhao, Q.; Luo, M.; Li, M.; Wang, D.; Wang, Y.; Liu, Q. Immobilization of Firefly Luciferase on PVA-Co-PE Nanofibers Membrane as Biosensor for Bioluminescent Detection of ATP. *ACS Appl. Mater. Interfaces* **2015**, *7*, 20046–20052.

(9) Cruz-Aguado, J. A.; Chen, Y.; Zhang, Z.; Elowe, N. H.; Brook, M. A.; Brennan, J. D. Ultrasensitive ATP Detection Using Firefly Luciferase Entrapped in Sugar-Modified Sol-Gel-Derived Silica. *J. Am. Chem. Soc.* **2004**, *126*, 6878–6879.

(10) Nowroozi-Nejad, Z.; Bahramian, B.; Hosseinkhani, S. Efficient Immobilization of Firefly Luciferase in a Metal Organic Framework: Fe-MIL-88(NH2) as a Mighty Support for This Purpose. *Enzyme Microb. Technol.* **2019**, *121*, 59–67.

(11) Nowroozi-Nejad, Z.; Bahramian, B.; Hosseinkhani, S. A Fast and Efficient Stabilization of Firefly Luciferase on MIL-53(Al) via Surface Adsorption Mechanism. *Res. Chem. Intermed.* **2019**, *45*, 2489–2501.

(12) Ebrahimi, M.; Hosseinkhani, S.; Heydari, A.; Akbari, J. Simple and Rapid Immobilization of Firefly Luciferase on Functionalized Magnetic Nanoparticles; a Try to Improve Kinetic Properties and Stability. *Biomacromol. J. Biomacromolecular J.* **2015**, *1*, 104–112.
